# Enrofloxacinium oxalate

**DOI:** 10.1107/S1600536814001421

**Published:** 2014-01-25

**Authors:** Thammarse S. Yamuna, Manpreet Kaur, Brian J. Anderson, Jerry P. Jasinski, H. S. Yathirajan

**Affiliations:** a Department of Studies in Chemistry, University of Mysore, Manasagangotri, Mysore 570 006, India; b Department of Chemistry, Keene State College, 229 Main Street, Keene, NH 03435-2001, USA

## Abstract

The title salt, 2C_19_H_23_FN_3_O_3_^+^·C_2_O_4_^2−^ {systematic name: bis-[4-(3-carb­oxy-1-cyclo­propyl-6-fluoro-4-oxo-1,4-di­hydro­quino­lin-7-yl)-1-ethyl­piperazin-1-ium] oxalate}, crystallizes with two independent monocations (*A* and *B*) and an oxalate dianion (*C*) in the asymmetric unit. The piperazinium ring in both the cations adopts a slightly disordered chair conformation. The dihedral angles between the mean planes of the cyclo­propyl ring and the 10-membered quinoline ring are 50.6 (5)° (*A*) and 62.2 (5)° (*B*). In each of the cations, a single O—H⋯O intra­molecular hydrogen bond is observed. In the crystal, the oxalate anions inter­act with the cations through N—H⋯O hydrogen bonds and weak C—H⋯O inter­actions, forming *R*_2_^2^(8) graph-set ring motifs. Weak C—H⋯F inter­actions along with further C—H⋯O inter­actions are observed between the cations, forming zigzag chains along [001]. In addition, π–π stacking inter­actions are observed with centroid–centroid distances of 3.5089 (13), 3.5583 (13), 3.7900 (13) and 3.7991 (13) Å.

## Related literature   

For general background and the pharmacological properties of fluoro­quinolines, see: Bhanot *et al.* (2001 ▶); Scholar (2003 ▶). For related structures of substituted fluorinated compounds, see: Golovnev *et al.* (2012 ▶); Harrison *et al.* (2007 ▶); Jasinski *et al.* (2011*a* ▶,*b* ▶); Kavitha *et al.* (2013 ▶); Maheswararao & Angshuman (2013 ▶); Recillas-Mota *et al.* (2007 ▶); Sun *et al.* (2004 ▶). Also, various salts of enfloxacin (Maheswararao & Angshuman, 2013 ▶) and enrofloxacinium citrate monohydrate (Golovnev *et al.*, 2012 ▶) have been reported. For puckering parameters, see Cremer & Pople (1975 ▶). For standard bond lengths, see: Allen *et al.* (1987 ▶). 
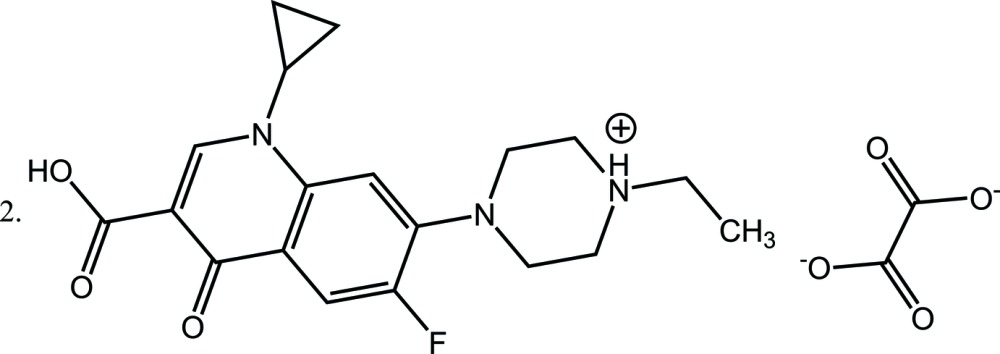


## Experimental   

### 

#### Crystal data  

2C_19_H_23_FN_3_O_3_^+^·C_2_O_4_^2−^
*M* *_r_* = 808.83
Triclinic, 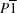

*a* = 9.8552 (5) Å
*b* = 13.3056 (8) Å
*c* = 15.6124 (8) Å
α = 68.987 (5)°
β = 84.740 (4)°
γ = 73.093 (5)°
*V* = 1828.31 (19) Å^3^
*Z* = 2
Cu *K*α radiation
μ = 0.95 mm^−1^
*T* = 173 K
0.24 × 0.16 × 0.08 mm

#### Data collection  

Agilent Xcalibur (Eos, Gemini) diffractometer
Absorption correction: multi-scan (*CrysAlis PRO* and *CrysAlis RED*; Agilent, 2012 ▶) *T*_min_ = 0.880, *T*_max_ = 1.000
11885 measured reflections
7017 independent reflections
5641 reflections with *I* > 2σ(*I*)
*R*_int_ = 0.024

#### Refinement  

*R*[*F* ^2^ > 2σ(*F* ^2^)] = 0.051
*wR*(*F* ^2^) = 0.145
*S* = 1.03
7017 reflections
527 parameters
H-atom parameters constrained
Δρ_max_ = 0.68 e Å^−3^
Δρ_min_ = −0.28 e Å^−3^

### 

Data collection: *CrysAlis PRO* (Agilent, 2012 ▶); cell refinement: *CrysAlis PRO*; data reduction: *CrysAlis RED* (Agilent, 2012 ▶); program(s) used to solve structure: *SUPERFLIP* (Palatinus & Chapuis, 2007 ▶); program(s) used to refine structure: *SHELXL2012* (Sheldrick, 2008 ▶); molecular graphics: *OLEX2* (Dolomanov *et al.*, 2009 ▶); software used to prepare material for publication: *OLEX2*.

## Supplementary Material

Crystal structure: contains datablock(s) I. DOI: 10.1107/S1600536814001421/hg5378sup1.cif

Structure factors: contains datablock(s) I. DOI: 10.1107/S1600536814001421/hg5378Isup2.hkl

Supporting information file. DOI: 10.1107/S1600536814001421/hg5378Isup3.cml

CCDC reference:

Additional supporting information:  crystallographic information; 3D view; checkCIF report

## Figures and Tables

**Table 1:** Hydrogen-bond geometry (Å, °)

*D*—H⋯*A*	*D*—H	H⋯*A*	*D*⋯*A*	*D*—H⋯*A*
O2*B*—H2*B*⋯O1*B*	0.82	1.78	2.542 (2)	154
N2*B*—H2*BA*⋯O2*C* ^i^	0.98	1.67	2.615 (2)	161
O2*A*—H2*A*⋯O1*A*	0.82	1.77	2.531 (2)	154
N2*A*—H2*AA*⋯O4*C* ^ii^	0.98	1.64	2.609 (2)	171
C10*B*—H10*B*⋯O1*C* ^i^	0.97	2.34	3.231 (3)	153
C11*B*—H11*A*⋯O1*C*	0.97	2.56	3.358 (3)	139
C12*B*—H12*A*⋯O3*B* ^iii^	0.97	2.51	3.302 (3)	138
C15*B*—H15*A*⋯O1*A* ^iii^	0.97	2.48	3.433 (3)	169
C16*B*—H16*A*⋯O3*B* ^iv^	0.97	2.46	3.167 (3)	130
C7*A*—H7*A*⋯F1*B* ^v^	0.93	2.54	3.314 (3)	141
C12*A*—H12*C*⋯O2*A* ^vi^	0.97	2.53	3.462 (3)	162
C13*A*—H13*C*⋯O3*C* ^ii^	0.97	2.47	3.254 (3)	137
C16*A*—H16*D*⋯O3*A* ^vii^	0.97	2.37	3.325 (3)	170
C18*A*—H18*D*⋯O1*C* ^viii^	0.97	2.58	3.236 (3)	125
C19*A*—H19*F*⋯O3*C* ^viii^	0.96	2.44	3.375 (3)	163

Symmetry codes: (i) 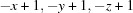; (ii) 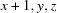; (iii) 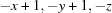; (iv) 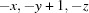; (v) 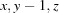; (vi) 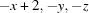; (vii) 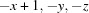; (viii) 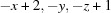.

**Fig. 1.**
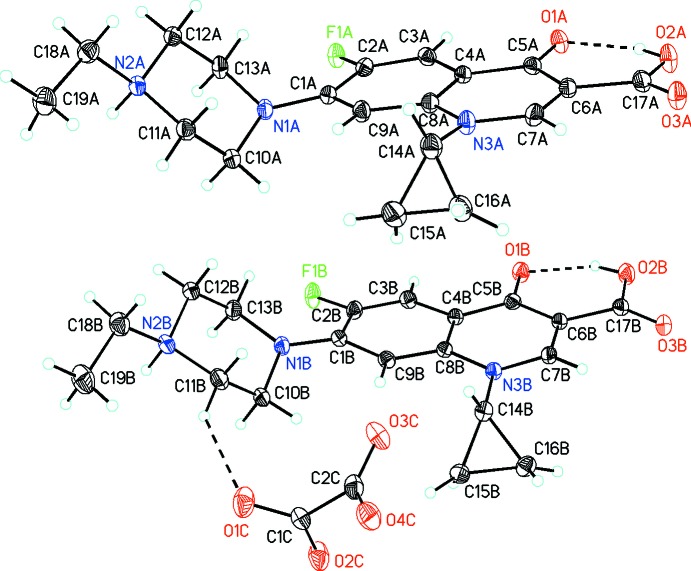
ORTEP drawing of (I) (2.(C19H23FO3N3+) . C2O42-) showing the labeling scheme with 30% probability displacement ellipsoids. Dashed lines indicate a O—H···O intramolecular hydrogen bond in the cations within the asymmetric unit.

**Fig. 2.**
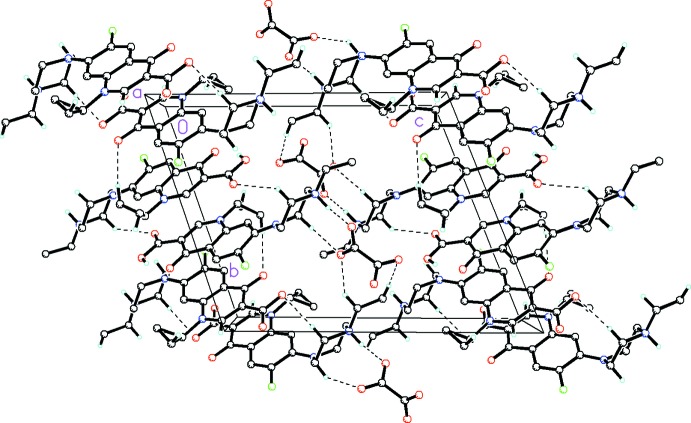
Molecular packing for (I) viewed along the a axis. Dashed lines indicate N—H···O intermolecular hydrogen bonds and weak C—H···O intermolecular interactions. H atoms not involved in hydrogen bonding have been removed for clarity.

## supplementary crystallographic information

### 1. Comment

Enrofloxacin [ systematic name : 1-Cyclopropyl-7-(4-ethyl-piperazin -1-yl)-6-fluoro-4-oxo-1,4-dihydro-quinoline-3-carboxylic acid is a fluoroquinolone antibiotic and is a synthetic chemotherapeutic agent from the class of the fluoroquinolone carboxylic acid derivatives. It is available under the trade name Baytril, from Bayer Corporation and has antibacterial activity against a broad spectrum of Gram-negative and Gram-positive bacteria. Its mechanism of action is not thoroughly understood, but it is believed to act by inhibiting bacterial DNA gyrase (a type-II topoisomerase), thereby preventing DNA supercoiling and DNA synthesis. The chemical and biological aspects of fluoroquinolones is described (Bhanot *et al.*, 2001; Scholar, 2003). Earlier, the crystal structure of the enrofloxacinium picrate (Jasinski *et al.*, 2011*a*), Flunarizinium hydrogen maleate (Kavitha *et al.*, 2013) and Lomefloxacinium picrate (Jasinski *et al.*, 2011*b*) have been reported by our group . The crystal structure of a copper complex of enrofloxacin (Recillas-Mota *et al.*, 2007), escitalopram oxalate: co-existence of oxalate dianions and oxalic acid molecules in the same crystal (Harrison *et al.*, 2007) and 2-hydroxyethanaminium enrofloxacinate (Sun *et al.*, 2004) have also been reported. Also, the crystal structures of various salts of enfloxacin (Maheswararao & Angshuman, 2013) and enrofloxacinium citrate monohydrate (Golovnev *et al.*, 2012) have been reported. In continuation of our work on substituted fluorinated compounds, this paper reports the crystal structure of the title salt, (I), 2(C_19_H_23_FO_3_N_3_^+^).C_2_O_4_^2-^.

The title salt, (I), 2(C_19_H_23_FO_3_N_3_^+^).C_2_O_4_^2-^, crystallizes with two independent monocations (A and B) and an oxalate dianion (C) in the asymmetric unit (Fig. 1). The piperazinium ring in both the cations adopts a slightly disordered chair conformation (puckering parameters (A) Q, θ, and φ = 0.560 (2)Å, 2.4 (2)° and 100 (5)°; (B) Q, θ, and φ = 0.563 (2)Å,4.5 (2)° and 172 (3)°, respectively; (Cremer & Pople, 1975). Bond lengths are in normal ranges (Allen *et al.*, 1987). The dihedral angles between the mean planes of the cyclopropyl ring and the 10-membered quinoline ring are 50.6 (5)° (A) and 62.2 (5)° (B), respectively. In the cations, a single O—H···O intramolecular hydrogen bond is observed. In the crystal, the oxalate anions interact with the cations through N—H···O intermolecular hydrogen bonds and weak C—H···O intermolecular interactions forming R_2_^2^(8) graph set ring motifs (Fig. 2). A weak C—H···F intermolecular interaction along with the C—H···O interactions are observed between the cations forming zig-zag chains. In addition, Cg—Cg π—π stacking interactions are observed which contribute to crystal packing stability (Cg3—Cg3 = 3.5583 (13)Å; Cg3—Cg4 = 3.7900 (13)Å; 2-x,-y,-z; Cg4—Cg8 = 3.7991 (13)Å; Cg8—Cg8 = 3.5089 (13)Å; 1-x,1-y,-z Cg3 = N3A/C7A/C6A/C5A/C4A/C8A; Cg4 = C1A–C9A; Cg8 = N3B/C7B/C6B/C5B/C4B/C8B).

### 2. Experimental

Gift sample from R. L. Fine Chemicals; enrofloxacin (0.6 g, 1.6 mmol) and oxalic acid (0.146 g, 1.6 mmol) were dissolved in a mixture of acetonitrile and dimethyl sulfoxide (DMSO) (4:1 v/v)and stirred at room temperature for 15 mins. The precipitate obtained was filtered, dried and dissolved in DMSO, stirred for 15 mins at 333 K. The solution was then allowed to cool at room temperature. After few days, X-ray quality crystals of the title compound were obtained by slow evaporation (m.p.: 498–503 K).

### 3. Refinement

All of the H atoms were placed in their calculated positions and then refined using the riding model with Atom—H lengths of 0.93Å (CH); 0.97Å (CH_2_); 0.96Å (CH_3_); 0.82Å (OH) or 0.98Å (NH) . Isotropic displacement parameters for these atoms were set to 1.2 (CH, CH_2_, NH) or 1.5 (CH_3_, OH) times *U*_eq_ of the parent atom. Idealised Me and tetrahedral OH were refined as rotating groups.

### Crystal data

**Table:**

2C_19_H_23_FN_3_O_3_^+^·C_2_O_4_^2^^−^	*Z* = 2
*M**_r_* = 808.83	*F*(000) = 852
Triclinic, *P*1	*D*_x_ = 1.469 Mg m^−^^3^
*a* = 9.8552 (5) Å	Cu *K*α radiation, λ = 1.54184 Å
*b* = 13.3056 (8) Å	Cell parameters from 4206 reflections
*c* = 15.6124 (8) Å	θ = 3.7–72.5°
α = 68.987 (5)°	µ = 0.95 mm^−^^1^
β = 84.740 (4)°	*T* = 173 K
γ = 73.093 (5)°	Irregular, colourless
*V* = 1828.31 (19) Å^3^	0.24 × 0.16 × 0.08 mm

### Data collection

**Table:**

Agilent Xcalibur (Eos, Gemini) diffractometer	7017 independent reflections
Radiation source: Enhance (Cu) X-ray Source	5641 reflections with *I* > 2σ(*I*)
Detector resolution: 16.0416 pixels mm^-1^	*R*_int_ = 0.024
ω scans	θ_max_ = 72.6°, θ_min_ = 3.7°
Absorption correction: multi-scan (*CrysAlis PRO* and *CrysAlis RED*; Agilent, 2012)	*h* = −12→10
*T*_min_ = 0.880, *T*_max_ = 1.000	*k* = −16→15
11885 measured reflections	*l* = −17→19

### Refinement

**Table:**

Refinement on *F*^2^	Primary atom site location: structure-invariant direct methods
Least-squares matrix: full	Hydrogen site location: inferred from neighbouring sites
*R*[*F*^2^ > 2σ(*F*^2^)] = 0.051	H-atom parameters constrained
*wR*(*F*^2^) = 0.145	*w* = 1/[σ^2^(*F*_o_^2^) + (0.0686*P*)^2^ + 1.1302*P*] where *P* = (*F*_o_^2^ + 2*F*_c_^2^)/3
*S* = 1.03	(Δ/σ)_max_ < 0.001
7017 reflections	Δρ_max_ = 0.68 e Å^−^^3^
527 parameters	Δρ_min_ = −0.28 e Å^−^^3^
0 restraints	

### Special details

**Table:**

Geometry. All esds (except the esd in the dihedral angle between two l.s. planes) are estimated using the full covariance matrix. The cell esds are taken into account individually in the estimation of esds in distances, angles and torsion angles; correlations between esds in cell parameters are only used when they are defined by crystal symmetry. An approximate (isotropic) treatment of cell esds is used for estimating esds involving l.s. planes.

### Fractional atomic coordinates and isotropic or equivalent isotropic displacement parameters (Å^2^)

**Table:**

	*x*	*y*	*z*	*U*_iso_*/*U*_eq_	
F1B	0.70509 (15)	0.74124 (11)	0.12480 (9)	0.0406 (3)	
O1B	0.45726 (17)	0.76166 (13)	−0.15035 (10)	0.0344 (4)	
O2B	0.30755 (18)	0.72055 (15)	−0.25107 (10)	0.0380 (4)	
H2B	0.3660	0.7429	−0.2343	0.057*	
O3B	0.16661 (17)	0.61191 (15)	−0.18637 (11)	0.0373 (4)	
N1B	0.64215 (19)	0.56582 (17)	0.27496 (12)	0.0317 (4)	
N2B	0.79517 (19)	0.43144 (15)	0.44580 (11)	0.0287 (4)	
H2BA	0.7737	0.4906	0.4725	0.034*	
N3B	0.31112 (18)	0.54720 (14)	0.07440 (11)	0.0245 (4)	
C1B	0.5793 (2)	0.60258 (17)	0.19053 (14)	0.0260 (4)	
C2B	0.6151 (2)	0.68662 (18)	0.11280 (14)	0.0280 (4)	
C3B	0.5611 (2)	0.71886 (17)	0.02720 (14)	0.0281 (4)	
H3B	0.5901	0.7734	−0.0211	0.034*	
C4B	0.4613 (2)	0.67010 (17)	0.01123 (13)	0.0246 (4)	
C5B	0.4119 (2)	0.69607 (17)	−0.08076 (14)	0.0261 (4)	
C6B	0.3120 (2)	0.63909 (17)	−0.08788 (14)	0.0258 (4)	
C7B	0.2664 (2)	0.56886 (17)	−0.01080 (14)	0.0266 (4)	
H7B	0.2008	0.5341	−0.0176	0.032*	
C8B	0.4148 (2)	0.59339 (16)	0.08783 (13)	0.0232 (4)	
C9B	0.4739 (2)	0.55956 (17)	0.17494 (13)	0.0254 (4)	
H9B	0.4425	0.5072	0.2238	0.030*	
C10B	0.5681 (2)	0.5247 (2)	0.36023 (14)	0.0336 (5)	
H10A	0.4814	0.5127	0.3463	0.040*	
H10B	0.5423	0.5809	0.3893	0.040*	
C11B	0.6586 (2)	0.4166 (2)	0.42578 (15)	0.0343 (5)	
H11A	0.6079	0.3933	0.4825	0.041*	
H11B	0.6777	0.3584	0.3991	0.041*	
C12B	0.8697 (2)	0.4687 (2)	0.35783 (14)	0.0316 (5)	
H12A	0.8919	0.4108	0.3306	0.038*	
H12B	0.9583	0.4795	0.3698	0.038*	
C13B	0.7800 (2)	0.5769 (2)	0.29089 (15)	0.0329 (5)	
H13A	0.7662	0.6368	0.3152	0.039*	
H13B	0.8292	0.5966	0.2331	0.039*	
C14B	0.2554 (2)	0.47290 (17)	0.15365 (14)	0.0273 (4)	
H14B	0.3176	0.3969	0.1816	0.033*	
C15B	0.1645 (2)	0.5239 (2)	0.21785 (15)	0.0322 (5)	
H15A	0.1454	0.6040	0.2032	0.039*	
H15B	0.1730	0.4800	0.2829	0.039*	
C16B	0.0994 (2)	0.48439 (19)	0.15630 (15)	0.0321 (5)	
H16A	0.0689	0.4168	0.1845	0.039*	
H16B	0.0413	0.5409	0.1047	0.039*	
C17B	0.2551 (2)	0.65490 (18)	−0.17814 (14)	0.0295 (5)	
C18B	0.8887 (3)	0.3272 (2)	0.51176 (16)	0.0391 (5)	
H18A	0.9804	0.3388	0.5154	0.047*	
H18B	0.9035	0.2663	0.4885	0.047*	
C19B	0.8275 (3)	0.2936 (2)	0.60727 (17)	0.0519 (7)	
H19A	0.7420	0.2737	0.6053	0.078*	
H19B	0.8065	0.3554	0.6290	0.078*	
H19C	0.8952	0.2306	0.6480	0.078*	
F1A	1.13064 (14)	0.29936 (11)	0.04417 (8)	0.0342 (3)	
O1A	0.85745 (17)	0.19630 (13)	−0.15083 (10)	0.0321 (3)	
O2A	0.68669 (19)	0.11660 (14)	−0.20006 (10)	0.0397 (4)	
H2A	0.7408	0.1535	−0.2009	0.060*	
O3A	0.60977 (18)	−0.01945 (14)	−0.09726 (11)	0.0391 (4)	
N1A	1.09798 (19)	0.17150 (16)	0.22569 (12)	0.0295 (4)	
N2A	1.27942 (18)	0.04206 (14)	0.38543 (11)	0.0253 (4)	
H2AA	1.2703	0.1003	0.4118	0.030*	
N3A	0.8099 (2)	0.00160 (16)	0.11512 (12)	0.0307 (4)	
C1A	1.0440 (2)	0.15549 (17)	0.15500 (14)	0.0262 (4)	
C2A	1.0595 (2)	0.22096 (17)	0.06202 (14)	0.0256 (4)	
C3A	0.9992 (2)	0.21296 (17)	−0.00905 (14)	0.0257 (4)	
H3A	1.0154	0.2555	−0.0689	0.031*	
C4A	0.9124 (2)	0.14100 (16)	0.00681 (13)	0.0238 (4)	
C5A	0.8418 (2)	0.13722 (17)	−0.06899 (14)	0.0252 (4)	
C6A	0.7540 (2)	0.06251 (17)	−0.04422 (14)	0.0263 (4)	
C7A	0.7432 (2)	−0.00186 (19)	0.04534 (15)	0.0298 (5)	
H7A	0.6867	−0.0506	0.0587	0.036*	
C8A	0.8943 (2)	0.07485 (17)	0.09788 (14)	0.0260 (4)	
C9A	0.9630 (2)	0.08076 (18)	0.16998 (14)	0.0290 (4)	
H9A	0.9543	0.0334	0.2295	0.035*	
C10A	1.0411 (2)	0.1374 (2)	0.31756 (14)	0.0322 (5)	
H10C	0.9480	0.1281	0.3142	0.039*	
H10D	1.0305	0.1959	0.3430	0.039*	
C11A	1.1360 (2)	0.02910 (19)	0.38023 (14)	0.0303 (5)	
H11C	1.0957	0.0094	0.4411	0.036*	
H11D	1.1429	−0.0307	0.3570	0.036*	
C12A	1.3383 (2)	0.07932 (18)	0.29130 (14)	0.0285 (4)	
H12C	1.3549	0.0200	0.2660	0.034*	
H12D	1.4288	0.0928	0.2952	0.034*	
C13A	1.2397 (2)	0.18483 (19)	0.22765 (14)	0.0302 (5)	
H13C	1.2334	0.2469	0.2480	0.036*	
H13D	1.2779	0.2023	0.1662	0.036*	
C14A	0.8049 (3)	−0.0800 (2)	0.20732 (15)	0.0351 (5)	
H14A	0.8879	−0.1454	0.2267	0.042*	
C15A	0.7333 (3)	−0.0368 (2)	0.28052 (17)	0.0419 (6)	
H15C	0.6916	0.0434	0.2638	0.050*	
H15D	0.7730	−0.0746	0.3422	0.050*	
C16A	0.6662 (3)	−0.0993 (2)	0.24179 (16)	0.0374 (5)	
H16C	0.6652	−0.1747	0.2802	0.045*	
H16D	0.5838	−0.0567	0.2018	0.045*	
C17A	0.6767 (2)	0.04869 (19)	−0.11462 (15)	0.0308 (5)	
C18A	1.3797 (2)	−0.06322 (19)	0.44442 (15)	0.0348 (5)	
H18C	1.4717	−0.0504	0.4445	0.042*	
H18D	1.3911	−0.1212	0.4183	0.042*	
C19A	1.3304 (3)	−0.1041 (2)	0.54241 (16)	0.0449 (6)	
H19D	1.2494	−0.1310	0.5441	0.067*	
H19E	1.3051	−0.0435	0.5657	0.067*	
H19F	1.4055	−0.1638	0.5796	0.067*	
O1C	0.4046 (2)	0.30357 (16)	0.54362 (14)	0.0533 (5)	
O2C	0.2052 (2)	0.42635 (15)	0.47408 (13)	0.0481 (5)	
O3C	0.3605 (2)	0.28420 (16)	0.35788 (12)	0.0496 (5)	
O4C	0.2434 (2)	0.18491 (15)	0.47003 (12)	0.0456 (4)	
C1C	0.3040 (2)	0.33643 (18)	0.48940 (15)	0.0317 (5)	
C2C	0.3017 (3)	0.26345 (19)	0.43358 (16)	0.0346 (5)	

### Atomic displacement parameters (Å^2^)

**Table:**

	*U*^11^	*U*^22^	*U*^33^	*U*^12^	*U*^13^	*U*^23^
F1B	0.0524 (8)	0.0426 (8)	0.0337 (7)	−0.0283 (7)	−0.0099 (6)	−0.0070 (6)
O1B	0.0380 (9)	0.0414 (9)	0.0221 (7)	−0.0177 (7)	−0.0020 (6)	−0.0032 (6)
O2B	0.0405 (9)	0.0505 (10)	0.0232 (8)	−0.0161 (8)	−0.0039 (6)	−0.0094 (7)
O3B	0.0346 (9)	0.0509 (10)	0.0337 (8)	−0.0138 (8)	−0.0040 (7)	−0.0205 (7)
N1B	0.0292 (9)	0.0466 (11)	0.0215 (9)	−0.0151 (8)	−0.0003 (7)	−0.0108 (8)
N2B	0.0354 (10)	0.0299 (9)	0.0225 (8)	−0.0077 (8)	−0.0013 (7)	−0.0117 (7)
N3B	0.0255 (9)	0.0258 (8)	0.0226 (8)	−0.0090 (7)	−0.0006 (6)	−0.0072 (7)
C1B	0.0262 (10)	0.0302 (10)	0.0233 (10)	−0.0068 (8)	0.0001 (8)	−0.0123 (8)
C2B	0.0284 (10)	0.0306 (11)	0.0297 (11)	−0.0137 (9)	−0.0012 (8)	−0.0113 (9)
C3B	0.0325 (11)	0.0280 (10)	0.0241 (10)	−0.0119 (9)	0.0017 (8)	−0.0068 (8)
C4B	0.0247 (10)	0.0253 (10)	0.0227 (10)	−0.0057 (8)	−0.0013 (8)	−0.0074 (8)
C5B	0.0251 (10)	0.0264 (10)	0.0245 (10)	−0.0046 (8)	0.0002 (8)	−0.0082 (8)
C6B	0.0239 (10)	0.0293 (10)	0.0241 (10)	−0.0039 (8)	−0.0023 (8)	−0.0113 (8)
C7B	0.0254 (10)	0.0293 (10)	0.0281 (10)	−0.0067 (8)	−0.0022 (8)	−0.0137 (8)
C8B	0.0229 (10)	0.0239 (9)	0.0237 (10)	−0.0053 (8)	0.0001 (7)	−0.0100 (8)
C9B	0.0284 (10)	0.0251 (10)	0.0221 (10)	−0.0087 (8)	0.0014 (8)	−0.0068 (8)
C10B	0.0308 (11)	0.0511 (14)	0.0227 (10)	−0.0146 (10)	0.0021 (8)	−0.0152 (10)
C11B	0.0402 (13)	0.0431 (13)	0.0251 (10)	−0.0184 (11)	0.0024 (9)	−0.0135 (10)
C12B	0.0291 (11)	0.0430 (13)	0.0272 (11)	−0.0121 (10)	0.0012 (8)	−0.0161 (9)
C13B	0.0328 (12)	0.0423 (13)	0.0269 (11)	−0.0165 (10)	−0.0024 (9)	−0.0105 (9)
C14B	0.0297 (11)	0.0244 (10)	0.0267 (10)	−0.0106 (8)	−0.0022 (8)	−0.0045 (8)
C15B	0.0330 (11)	0.0365 (12)	0.0263 (10)	−0.0130 (9)	0.0032 (8)	−0.0080 (9)
C16B	0.0297 (11)	0.0317 (11)	0.0330 (11)	−0.0121 (9)	−0.0004 (9)	−0.0059 (9)
C17B	0.0260 (10)	0.0347 (11)	0.0276 (11)	−0.0027 (9)	−0.0030 (8)	−0.0144 (9)
C18B	0.0461 (14)	0.0344 (12)	0.0332 (12)	−0.0036 (11)	−0.0066 (10)	−0.0116 (10)
C19B	0.0691 (19)	0.0451 (15)	0.0316 (13)	−0.0115 (14)	−0.0073 (12)	−0.0035 (11)
F1A	0.0425 (7)	0.0353 (7)	0.0285 (6)	−0.0195 (6)	−0.0064 (5)	−0.0068 (5)
O1A	0.0425 (9)	0.0348 (8)	0.0214 (7)	−0.0150 (7)	−0.0033 (6)	−0.0083 (6)
O2A	0.0536 (11)	0.0451 (10)	0.0260 (8)	−0.0232 (8)	−0.0085 (7)	−0.0093 (7)
O3A	0.0459 (10)	0.0476 (10)	0.0330 (8)	−0.0240 (8)	−0.0051 (7)	−0.0145 (7)
N1A	0.0279 (9)	0.0415 (10)	0.0221 (8)	−0.0124 (8)	−0.0026 (7)	−0.0117 (8)
N2A	0.0277 (9)	0.0260 (9)	0.0234 (8)	−0.0072 (7)	−0.0034 (7)	−0.0093 (7)
N3A	0.0338 (10)	0.0371 (10)	0.0226 (9)	−0.0157 (8)	−0.0037 (7)	−0.0063 (7)
C1A	0.0238 (10)	0.0302 (10)	0.0240 (10)	−0.0040 (8)	−0.0045 (8)	−0.0103 (8)
C2A	0.0238 (10)	0.0258 (10)	0.0282 (10)	−0.0083 (8)	−0.0017 (8)	−0.0091 (8)
C3A	0.0291 (10)	0.0243 (10)	0.0215 (9)	−0.0056 (8)	−0.0001 (8)	−0.0068 (8)
C4A	0.0234 (10)	0.0247 (10)	0.0232 (10)	−0.0044 (8)	−0.0016 (7)	−0.0093 (8)
C5A	0.0256 (10)	0.0247 (10)	0.0240 (10)	−0.0026 (8)	−0.0012 (8)	−0.0101 (8)
C6A	0.0268 (10)	0.0272 (10)	0.0259 (10)	−0.0050 (8)	−0.0042 (8)	−0.0113 (8)
C7A	0.0282 (11)	0.0339 (11)	0.0304 (11)	−0.0132 (9)	−0.0027 (8)	−0.0106 (9)
C8A	0.0259 (10)	0.0292 (10)	0.0236 (10)	−0.0088 (8)	−0.0018 (8)	−0.0086 (8)
C9A	0.0299 (11)	0.0355 (11)	0.0203 (10)	−0.0110 (9)	−0.0024 (8)	−0.0061 (8)
C10A	0.0271 (11)	0.0462 (13)	0.0248 (10)	−0.0083 (10)	−0.0008 (8)	−0.0154 (9)
C11A	0.0303 (11)	0.0389 (12)	0.0257 (10)	−0.0156 (9)	0.0014 (8)	−0.0114 (9)
C12A	0.0252 (10)	0.0370 (11)	0.0264 (10)	−0.0111 (9)	0.0008 (8)	−0.0130 (9)
C13A	0.0344 (11)	0.0360 (11)	0.0235 (10)	−0.0164 (9)	−0.0044 (8)	−0.0080 (9)
C14A	0.0391 (13)	0.0370 (12)	0.0282 (11)	−0.0130 (10)	−0.0025 (9)	−0.0077 (9)
C15A	0.0470 (14)	0.0453 (14)	0.0358 (13)	−0.0150 (12)	0.0017 (11)	−0.0151 (11)
C16A	0.0419 (13)	0.0446 (13)	0.0299 (11)	−0.0201 (11)	0.0025 (10)	−0.0119 (10)
C17A	0.0328 (11)	0.0335 (11)	0.0285 (11)	−0.0078 (9)	−0.0034 (9)	−0.0137 (9)
C18A	0.0353 (12)	0.0306 (11)	0.0332 (12)	−0.0039 (9)	−0.0049 (9)	−0.0077 (9)
C19A	0.0518 (15)	0.0406 (14)	0.0308 (12)	−0.0068 (12)	−0.0062 (11)	−0.0022 (10)
O1C	0.0546 (12)	0.0499 (11)	0.0629 (12)	−0.0027 (9)	−0.0203 (10)	−0.0325 (10)
O2C	0.0559 (11)	0.0402 (10)	0.0513 (11)	0.0029 (8)	−0.0187 (9)	−0.0278 (8)
O3C	0.0676 (13)	0.0535 (11)	0.0318 (9)	−0.0168 (10)	0.0009 (8)	−0.0197 (8)
O4C	0.0602 (12)	0.0431 (10)	0.0428 (10)	−0.0201 (9)	0.0007 (8)	−0.0214 (8)
C1C	0.0404 (12)	0.0304 (11)	0.0261 (10)	−0.0096 (10)	−0.0008 (9)	−0.0118 (9)
C2C	0.0383 (12)	0.0321 (12)	0.0319 (12)	−0.0035 (10)	−0.0099 (9)	−0.0117 (9)

### Geometric parameters (Å, º)

**Table:**

F1B—C2B	1.362 (2)	O2A—C17A	1.330 (3)
O1B—C5B	1.265 (2)	O3A—C17A	1.212 (3)
O2B—H2B	0.8200	N1A—C1A	1.374 (3)
O2B—C17B	1.330 (3)	N1A—C10A	1.453 (3)
O3B—C17B	1.212 (3)	N1A—C13A	1.463 (3)
N1B—C1B	1.368 (3)	N2A—H2AA	0.9800
N1B—C10B	1.462 (3)	N2A—C11A	1.485 (3)
N1B—C13B	1.461 (3)	N2A—C12A	1.494 (3)
N2B—H2BA	0.9800	N2A—C18A	1.491 (3)
N2B—C11B	1.492 (3)	N3A—C7A	1.344 (3)
N2B—C12B	1.487 (3)	N3A—C8A	1.399 (3)
N2B—C18B	1.497 (3)	N3A—C14A	1.466 (3)
N3B—C7B	1.344 (3)	C1A—C2A	1.421 (3)
N3B—C8B	1.401 (3)	C1A—C9A	1.393 (3)
N3B—C14B	1.457 (2)	C2A—C3A	1.356 (3)
C1B—C2B	1.423 (3)	C3A—H3A	0.9300
C1B—C9B	1.399 (3)	C3A—C4A	1.406 (3)
C2B—C3B	1.356 (3)	C4A—C5A	1.449 (3)
C3B—H3B	0.9300	C4A—C8A	1.405 (3)
C3B—C4B	1.408 (3)	C5A—C6A	1.432 (3)
C4B—C5B	1.447 (3)	C6A—C7A	1.366 (3)
C4B—C8B	1.406 (3)	C6A—C17A	1.484 (3)
C5B—C6B	1.439 (3)	C7A—H7A	0.9300
C6B—C7B	1.365 (3)	C8A—C9A	1.402 (3)
C6B—C17B	1.486 (3)	C9A—H9A	0.9300
C7B—H7B	0.9300	C10A—H10C	0.9700
C8B—C9B	1.394 (3)	C10A—H10D	0.9700
C9B—H9B	0.9300	C10A—C11A	1.510 (3)
C10B—H10A	0.9700	C11A—H11C	0.9700
C10B—H10B	0.9700	C11A—H11D	0.9700
C10B—C11B	1.509 (3)	C12A—H12C	0.9700
C11B—H11A	0.9700	C12A—H12D	0.9700
C11B—H11B	0.9700	C12A—C13A	1.512 (3)
C12B—H12A	0.9700	C13A—H13C	0.9700
C12B—H12B	0.9700	C13A—H13D	0.9700
C12B—C13B	1.516 (3)	C14A—H14A	0.9800
C13B—H13A	0.9700	C14A—C15A	1.490 (3)
C13B—H13B	0.9700	C14A—C16A	1.481 (3)
C14B—H14B	0.9800	C15A—H15C	0.9700
C14B—C15B	1.495 (3)	C15A—H15D	0.9700
C14B—C16B	1.499 (3)	C15A—C16A	1.498 (3)
C15B—H15A	0.9700	C16A—H16C	0.9700
C15B—H15B	0.9700	C16A—H16D	0.9700
C15B—C16B	1.511 (3)	C18A—H18C	0.9700
C16B—H16A	0.9700	C18A—H18D	0.9700
C16B—H16B	0.9700	C18A—C19A	1.514 (3)
C18B—H18A	0.9700	C19A—H19D	0.9600
C18B—H18B	0.9700	C19A—H19E	0.9600
C18B—C19B	1.517 (4)	C19A—H19F	0.9600
C19B—H19A	0.9600	O1C—C1C	1.235 (3)
C19B—H19B	0.9600	O2C—C1C	1.264 (3)
C19B—H19C	0.9600	O3C—C2C	1.242 (3)
F1A—C2A	1.356 (2)	O4C—C2C	1.268 (3)
O1A—C5A	1.260 (2)	C1C—C2C	1.525 (3)
O2A—H2A	0.8200		
			
C17B—O2B—H2B	109.5	C10A—N1A—C13A	111.08 (16)
C1B—N1B—C10B	122.36 (18)	C11A—N2A—H2AA	108.2
C1B—N1B—C13B	124.61 (18)	C11A—N2A—C12A	109.96 (15)
C13B—N1B—C10B	112.54 (17)	C11A—N2A—C18A	112.47 (17)
C11B—N2B—H2BA	108.4	C12A—N2A—H2AA	108.2
C11B—N2B—C18B	112.92 (18)	C18A—N2A—H2AA	108.2
C12B—N2B—H2BA	108.4	C18A—N2A—C12A	109.69 (16)
C12B—N2B—C11B	108.48 (16)	C7A—N3A—C8A	119.97 (18)
C12B—N2B—C18B	110.20 (17)	C7A—N3A—C14A	119.13 (18)
C18B—N2B—H2BA	108.4	C8A—N3A—C14A	120.60 (17)
C7B—N3B—C8B	120.31 (17)	N1A—C1A—C2A	121.21 (19)
C7B—N3B—C14B	120.42 (17)	N1A—C1A—C9A	122.43 (19)
C8B—N3B—C14B	119.24 (16)	C9A—C1A—C2A	116.21 (18)
N1B—C1B—C2B	123.04 (19)	F1A—C2A—C1A	118.45 (17)
N1B—C1B—C9B	121.74 (19)	F1A—C2A—C3A	118.63 (18)
C9B—C1B—C2B	115.22 (18)	C3A—C2A—C1A	122.80 (19)
F1B—C2B—C1B	118.60 (18)	C2A—C3A—H3A	119.7
C3B—C2B—F1B	117.39 (18)	C2A—C3A—C4A	120.68 (19)
C3B—C2B—C1B	123.97 (19)	C4A—C3A—H3A	119.7
C2B—C3B—H3B	119.9	C3A—C4A—C5A	120.52 (18)
C2B—C3B—C4B	120.21 (19)	C8A—C4A—C3A	118.19 (18)
C4B—C3B—H3B	119.9	C8A—C4A—C5A	121.28 (18)
C3B—C4B—C5B	121.03 (18)	O1A—C5A—C4A	121.67 (19)
C8B—C4B—C3B	117.31 (18)	O1A—C5A—C6A	122.88 (18)
C8B—C4B—C5B	121.64 (18)	C6A—C5A—C4A	115.45 (18)
O1B—C5B—C4B	122.03 (19)	C5A—C6A—C17A	121.50 (18)
O1B—C5B—C6B	122.41 (19)	C7A—C6A—C5A	120.70 (18)
C6B—C5B—C4B	115.52 (18)	C7A—C6A—C17A	117.75 (19)
C5B—C6B—C17B	121.59 (19)	N3A—C7A—C6A	123.4 (2)
C7B—C6B—C5B	120.28 (18)	N3A—C7A—H7A	118.3
C7B—C6B—C17B	118.13 (19)	C6A—C7A—H7A	118.3
N3B—C7B—C6B	123.55 (19)	N3A—C8A—C4A	119.14 (18)
N3B—C7B—H7B	118.2	N3A—C8A—C9A	120.75 (18)
C6B—C7B—H7B	118.2	C9A—C8A—C4A	120.09 (19)
N3B—C8B—C4B	118.48 (17)	C1A—C9A—C8A	121.89 (19)
C9B—C8B—N3B	120.03 (18)	C1A—C9A—H9A	119.1
C9B—C8B—C4B	121.43 (18)	C8A—C9A—H9A	119.1
C1B—C9B—H9B	119.2	N1A—C10A—H10C	109.2
C8B—C9B—C1B	121.55 (19)	N1A—C10A—H10D	109.2
C8B—C9B—H9B	119.2	N1A—C10A—C11A	111.86 (18)
N1B—C10B—H10A	109.3	H10C—C10A—H10D	107.9
N1B—C10B—H10B	109.3	C11A—C10A—H10C	109.2
N1B—C10B—C11B	111.66 (19)	C11A—C10A—H10D	109.2
H10A—C10B—H10B	107.9	N2A—C11A—C10A	109.78 (17)
C11B—C10B—H10A	109.3	N2A—C11A—H11C	109.7
C11B—C10B—H10B	109.3	N2A—C11A—H11D	109.7
N2B—C11B—C10B	110.48 (18)	C10A—C11A—H11C	109.7
N2B—C11B—H11A	109.6	C10A—C11A—H11D	109.7
N2B—C11B—H11B	109.6	H11C—C11A—H11D	108.2
C10B—C11B—H11A	109.6	N2A—C12A—H12C	109.2
C10B—C11B—H11B	109.6	N2A—C12A—H12D	109.2
H11A—C11B—H11B	108.1	N2A—C12A—C13A	112.13 (17)
N2B—C12B—H12A	109.3	H12C—C12A—H12D	107.9
N2B—C12B—H12B	109.3	C13A—C12A—H12C	109.2
N2B—C12B—C13B	111.51 (18)	C13A—C12A—H12D	109.2
H12A—C12B—H12B	108.0	N1A—C13A—C12A	111.11 (17)
C13B—C12B—H12A	109.3	N1A—C13A—H13C	109.4
C13B—C12B—H12B	109.3	N1A—C13A—H13D	109.4
N1B—C13B—C12B	111.01 (18)	C12A—C13A—H13C	109.4
N1B—C13B—H13A	109.4	C12A—C13A—H13D	109.4
N1B—C13B—H13B	109.4	H13C—C13A—H13D	108.0
C12B—C13B—H13A	109.4	N3A—C14A—H14A	116.2
C12B—C13B—H13B	109.4	N3A—C14A—C15A	118.0 (2)
H13A—C13B—H13B	108.0	N3A—C14A—C16A	118.3 (2)
N3B—C14B—H14B	116.3	C15A—C14A—H14A	116.2
N3B—C14B—C15B	117.44 (17)	C16A—C14A—H14A	116.2
N3B—C14B—C16B	118.40 (17)	C16A—C14A—C15A	60.58 (16)
C15B—C14B—H14B	116.3	C14A—C15A—H15C	117.8
C15B—C14B—C16B	60.61 (14)	C14A—C15A—H15D	117.8
C16B—C14B—H14B	116.3	C14A—C15A—C16A	59.40 (16)
C14B—C15B—H15A	117.8	H15C—C15A—H15D	115.0
C14B—C15B—H15B	117.8	C16A—C15A—H15C	117.8
C14B—C15B—C16B	59.81 (14)	C16A—C15A—H15D	117.8
H15A—C15B—H15B	114.9	C14A—C16A—C15A	60.02 (16)
C16B—C15B—H15A	117.8	C14A—C16A—H16C	117.8
C16B—C15B—H15B	117.8	C14A—C16A—H16D	117.8
C14B—C16B—C15B	59.58 (14)	C15A—C16A—H16C	117.8
C14B—C16B—H16A	117.8	C15A—C16A—H16D	117.8
C14B—C16B—H16B	117.8	H16C—C16A—H16D	114.9
C15B—C16B—H16A	117.8	O2A—C17A—C6A	115.03 (19)
C15B—C16B—H16B	117.8	O3A—C17A—O2A	121.40 (19)
H16A—C16B—H16B	115.0	O3A—C17A—C6A	123.6 (2)
O2B—C17B—C6B	115.55 (18)	N2A—C18A—H18C	109.0
O3B—C17B—O2B	121.15 (19)	N2A—C18A—H18D	109.0
O3B—C17B—C6B	123.3 (2)	N2A—C18A—C19A	113.00 (19)
N2B—C18B—H18A	109.0	H18C—C18A—H18D	107.8
N2B—C18B—H18B	109.0	C19A—C18A—H18C	109.0
N2B—C18B—C19B	112.9 (2)	C19A—C18A—H18D	109.0
H18A—C18B—H18B	107.8	C18A—C19A—H19D	109.5
C19B—C18B—H18A	109.0	C18A—C19A—H19E	109.5
C19B—C18B—H18B	109.0	C18A—C19A—H19F	109.5
C18B—C19B—H19A	109.5	H19D—C19A—H19E	109.5
C18B—C19B—H19B	109.5	H19D—C19A—H19F	109.5
C18B—C19B—H19C	109.5	H19E—C19A—H19F	109.5
H19A—C19B—H19B	109.5	O1C—C1C—O2C	126.0 (2)
H19A—C19B—H19C	109.5	O1C—C1C—C2C	116.9 (2)
H19B—C19B—H19C	109.5	O2C—C1C—C2C	117.03 (19)
C17A—O2A—H2A	109.5	O3C—C2C—O4C	126.0 (2)
C1A—N1A—C10A	120.94 (18)	O3C—C2C—C1C	117.1 (2)
C1A—N1A—C13A	124.69 (18)	O4C—C2C—C1C	116.9 (2)
			
F1B—C2B—C3B—C4B	−176.45 (19)	O1A—C5A—C6A—C17A	1.0 (3)
O1B—C5B—C6B—C7B	179.8 (2)	N1A—C1A—C2A—F1A	0.7 (3)
O1B—C5B—C6B—C17B	−0.5 (3)	N1A—C1A—C2A—C3A	−175.23 (19)
N1B—C1B—C2B—F1B	−7.0 (3)	N1A—C1A—C9A—C8A	172.2 (2)
N1B—C1B—C2B—C3B	175.4 (2)	N1A—C10A—C11A—N2A	58.6 (2)
N1B—C1B—C9B—C8B	−176.91 (19)	N2A—C12A—C13A—N1A	−54.1 (2)
N1B—C10B—C11B—N2B	56.9 (2)	N3A—C8A—C9A—C1A	−177.9 (2)
N2B—C12B—C13B—N1B	−55.8 (2)	N3A—C14A—C15A—C16A	108.5 (2)
N3B—C8B—C9B—C1B	178.95 (18)	N3A—C14A—C16A—C15A	−107.9 (2)
N3B—C14B—C15B—C16B	108.8 (2)	C1A—N1A—C10A—C11A	102.4 (2)
N3B—C14B—C16B—C15B	−107.3 (2)	C1A—N1A—C13A—C12A	−104.5 (2)
C1B—N1B—C10B—C11B	133.6 (2)	C1A—C2A—C3A—C4A	2.5 (3)
C1B—N1B—C13B—C12B	−134.9 (2)	C2A—C1A—C9A—C8A	−3.4 (3)
C1B—C2B—C3B—C4B	1.2 (3)	C2A—C3A—C4A—C5A	176.61 (19)
C2B—C1B—C9B—C8B	3.3 (3)	C2A—C3A—C4A—C8A	−2.3 (3)
C2B—C3B—C4B—C5B	−174.5 (2)	C3A—C4A—C5A—O1A	1.1 (3)
C2B—C3B—C4B—C8B	3.9 (3)	C3A—C4A—C5A—C6A	−178.78 (18)
C3B—C4B—C5B—O1B	1.1 (3)	C3A—C4A—C8A—N3A	−179.17 (18)
C3B—C4B—C5B—C6B	178.88 (19)	C3A—C4A—C8A—C9A	−0.6 (3)
C3B—C4B—C8B—N3B	177.35 (18)	C4A—C5A—C6A—C7A	−1.7 (3)
C3B—C4B—C8B—C9B	−5.4 (3)	C4A—C5A—C6A—C17A	−179.16 (18)
C4B—C5B—C6B—C7B	2.1 (3)	C4A—C8A—C9A—C1A	3.6 (3)
C4B—C5B—C6B—C17B	−178.24 (18)	C5A—C4A—C8A—N3A	1.9 (3)
C4B—C8B—C9B—C1B	1.7 (3)	C5A—C4A—C8A—C9A	−179.53 (19)
C5B—C4B—C8B—N3B	−4.2 (3)	C5A—C6A—C7A—N3A	1.4 (3)
C5B—C4B—C8B—C9B	173.05 (18)	C5A—C6A—C17A—O2A	−5.0 (3)
C5B—C6B—C7B—N3B	−0.9 (3)	C5A—C6A—C17A—O3A	174.4 (2)
C5B—C6B—C17B—O2B	3.0 (3)	C7A—N3A—C8A—C4A	−2.4 (3)
C5B—C6B—C17B—O3B	−176.8 (2)	C7A—N3A—C8A—C9A	179.1 (2)
C7B—N3B—C8B—C4B	5.5 (3)	C7A—N3A—C14A—C15A	−116.6 (2)
C7B—N3B—C8B—C9B	−171.83 (18)	C7A—N3A—C14A—C16A	−46.8 (3)
C7B—N3B—C14B—C15B	−112.4 (2)	C7A—C6A—C17A—O2A	177.5 (2)
C7B—N3B—C14B—C16B	−42.8 (3)	C7A—C6A—C17A—O3A	−3.1 (3)
C7B—C6B—C17B—O2B	−177.35 (19)	C8A—N3A—C7A—C6A	0.8 (3)
C7B—C6B—C17B—O3B	2.8 (3)	C8A—N3A—C14A—C15A	69.7 (3)
C8B—N3B—C7B—C6B	−3.0 (3)	C8A—N3A—C14A—C16A	139.5 (2)
C8B—N3B—C14B—C15B	69.3 (2)	C8A—C4A—C5A—O1A	179.98 (19)
C8B—N3B—C14B—C16B	138.96 (19)	C8A—C4A—C5A—C6A	0.1 (3)
C8B—C4B—C5B—O1B	−177.22 (19)	C9A—C1A—C2A—F1A	176.35 (18)
C8B—C4B—C5B—C6B	0.5 (3)	C9A—C1A—C2A—C3A	0.4 (3)
C9B—C1B—C2B—F1B	172.84 (18)	C10A—N1A—C1A—C2A	157.2 (2)
C9B—C1B—C2B—C3B	−4.8 (3)	C10A—N1A—C1A—C9A	−18.1 (3)
C10B—N1B—C1B—C2B	150.0 (2)	C10A—N1A—C13A—C12A	55.0 (2)
C10B—N1B—C1B—C9B	−29.8 (3)	C11A—N2A—C12A—C13A	54.7 (2)
C10B—N1B—C13B—C12B	53.0 (3)	C11A—N2A—C18A—C19A	−59.7 (2)
C11B—N2B—C12B—C13B	58.3 (2)	C12A—N2A—C11A—C10A	−55.9 (2)
C11B—N2B—C18B—C19B	−66.5 (3)	C12A—N2A—C18A—C19A	177.63 (19)
C12B—N2B—C11B—C10B	−58.4 (2)	C13A—N1A—C1A—C2A	−45.1 (3)
C12B—N2B—C18B—C19B	172.0 (2)	C13A—N1A—C1A—C9A	139.5 (2)
C13B—N1B—C1B—C2B	−21.4 (3)	C13A—N1A—C10A—C11A	−58.0 (2)
C13B—N1B—C1B—C9B	158.8 (2)	C14A—N3A—C7A—C6A	−172.9 (2)
C13B—N1B—C10B—C11B	−54.1 (2)	C14A—N3A—C8A—C4A	171.22 (19)
C14B—N3B—C7B—C6B	178.77 (19)	C14A—N3A—C8A—C9A	−7.3 (3)
C14B—N3B—C8B—C4B	−176.27 (18)	C17A—C6A—C7A—N3A	178.9 (2)
C14B—N3B—C8B—C9B	6.4 (3)	C18A—N2A—C11A—C10A	−178.43 (17)
C17B—C6B—C7B—N3B	179.40 (18)	C18A—N2A—C12A—C13A	178.91 (17)
C18B—N2B—C11B—C10B	179.11 (17)	O1C—C1C—C2C—O3C	−90.8 (3)
C18B—N2B—C12B—C13B	−177.60 (18)	O1C—C1C—C2C—O4C	87.1 (3)
F1A—C2A—C3A—C4A	−173.48 (18)	O2C—C1C—C2C—O3C	87.3 (3)
O1A—C5A—C6A—C7A	178.4 (2)	O2C—C1C—C2C—O4C	−94.8 (3)

### Hydrogen-bond geometry (Å, º)

**Table:**

*D*—H···*A*	*D*—H	H···*A*	*D*···*A*	*D*—H···*A*
O2*B*—H2*B*···O1*B*	0.82	1.78	2.542 (2)	154
N2*B*—H2*BA*···O2*C*^i^	0.98	1.67	2.615 (2)	161
O2*A*—H2*A*···O1*A*	0.82	1.77	2.531 (2)	154
N2*A*—H2*AA*···O4*C*^ii^	0.98	1.64	2.609 (2)	171
C10*B*—H10*B*···O1*C*^i^	0.97	2.34	3.231 (3)	153
C11*B*—H11*A*···O1*C*	0.97	2.56	3.358 (3)	139
C12*B*—H12*A*···O3*B*^iii^	0.97	2.51	3.302 (3)	138
C15*B*—H15*A*···O1*A*^iii^	0.97	2.48	3.433 (3)	169
C16*B*—H16*A*···O3*B*^iv^	0.97	2.46	3.167 (3)	130
C7*A*—H7*A*···F1*B*^v^	0.93	2.54	3.314 (3)	141
C12*A*—H12*C*···O2*A*^vi^	0.97	2.53	3.462 (3)	162
C13*A*—H13*C*···O3*C*^ii^	0.97	2.47	3.254 (3)	137
C16*A*—H16*D*···O3*A*^vii^	0.97	2.37	3.325 (3)	170
C18*A*—H18*D*···O1*C*^viii^	0.97	2.58	3.236 (3)	125
C19*A*—H19*F*···O3*C*^viii^	0.96	2.44	3.375 (3)	163

Symmetry codes: (i) −*x*+1, −*y*+1, −*z*+1; (ii) *x*+1, *y*, *z*; (iii) −*x*+1, −*y*+1, −*z*; (iv) −*x*, −*y*+1, −*z*; (v) *x*, *y*−1, *z*; (vi) −*x*+2, −*y*, −*z*; (vii) −*x*+1, −*y*, −*z*; (viii) −*x*+2, −*y*, −*z*+1.
